# The role of double-balloon enteroscopy following capsule endoscopy in diagnosis of obscure Small intestinal diseases

**DOI:** 10.12669/pjms.292.2927

**Published:** 2013-04

**Authors:** Chen Tian Min, Xu Li Hua, Ji Ying Lin, Yang Yan Mei, Lu Fei, Qian Jun Bo

**Affiliations:** 1 Chen Tian Min, Department of Gastroenterology, The first people’s Hospital of Nantong, Jiangsu, China 226001.; 2 Xu Li Hua, Department of Gastroenterology, The first people’s Hospital of Nantong, Jiangsu, China 226001.; 3 Ji Ying Lin, Department of Gastroenterology, The first people’s Hospital of Nantong, Jiangsu, China 226001.; 4 Yang Yan Mei, Department of Gastroenterology, The first people’s Hospital of Nantong, Jiangsu, China 226001.; 5 Lu Fei, Department of Gastroenterology, The first people’s Hospital of Nantong, Jiangsu, China 226001.; 6 Qian Jun Bo, Department of Gastroenterology, The first people’s Hospital of Nantong, Jiangsu, China 226001.

**Keywords:** Capsule Endoscopy, Double-Balloon Enteroscopy, Small Intestinal Disease, Diagnosis

## Abstract

***Objective:*** The aim of this study was to evaluate the detection rate accuracy of Double-balloon Enteroscopy (DBE) after Capsule Endoscopy (CE) in patients with suspected small bowel diseases.

**Methodology:** From January 2009 to March 2012, sixty-two patients with obscure small bowel diseases who underwent CE followed by DBE were included in this study. Introduction of the endoscope by DBE was either orally or anally according to CE.

***Results:*** Sixty-two patients are reported. The overall detection rate of small bowel diseases using CE was 70.9% (44/62). Sixty-eight DBE procedures following capsule endoscopy were carried out, There was no significant difference (χ^2^=0.6739, P>0.05) of Positive findings between CE and CE +DBE. Furthermore, the detection rate of small bowel diseases in patients with obscure small intestinal bleeding using CE +DBE (90.9%, 30/33) was superior to that of CE (78.8%, 26/33); χ^2^=1.8857, P>0.05.

***Conclusions:*** Capsule Endoscopy (CE) can cover the whole GI tract and provide the selection of the route of Double-balloon enteroscopy (DBE). DBE can also serve as a good complementary approach after an initial imaging using CE. It can verify the findings of CE and provide therapeutic intervention. Using of CE followed by DBE is effective in the diagnosis and management of patients with obscure small bowel diseases.

## INTRODUCTION

Small intestine is the middle part of digestive tract, due to its anatomic location, structural characteristics, and physiological functions, it is difficult to inspect via gastroscopy and colonoscopy. The diagnostic yield and accuracy of conventional diagnostic strategies including small intestine radiography, abdominal computed tomography (CT), magnetic resonance imaging (MRI), digital subtraction angiography (DSA), radionuclide, and intraoperative endoscopy is not satisfactory for detecting small intestine diseases.^1^^-^3 Capsule endoscopy (CE) and double-balloon enteroscopy (DBE) are two novel methods in the field of diagnosing and managing small bowel lesions.^4^^-^^9^ However, the role of these newer tests is still likely to be indefinite. It has been reported that the overall diagnostic yield of CE and DBE in patients with suspected small bowel disorders were 87% and 76%, respectively.^10^^,^^11^

The purpose of this study was to evaluate DBE following CE in patients with small bowel diseases through the retrospective study of CE and DBE.

## METHODOLOGY

***Patients: ***From January 2009 to March 2012, there were 253 patients using CE in our hospitals, sixty-two patients (28 women) with an average age of 55.4 years (range 23 to 78 years) in whom it had not been possible to determine the causes of small bowel bleeding, abdominal pain or diarrhea using conventional diagnostic procedures, such as gastroscopy, colonoscopy, radiological small bowel follow-throughs, enteroclysis, angiography, scintigraphy, computed tomography (CT) or magnetic resonance imaging (MRI) scans of the abdomen used in some patients were enrolled in the study. The duration of symptoms ranged 20 days—over 20 years. The main characteristics of patients are shown in Table-I. All the patients evaluated underwent CE followed by DBE. CE anteceded DBE by a median of 15 days (range, 4-60 days). All the procedures were performed after obtaining the informed consent from the patients, which was approved by the Hospitals’ Ethics Committee.

***Protocol: ***Patients with contraindications to CE such as gastrointestinal obstruction, stricture or fistula, cardiac pacemakers, other implanted electromedical devices, swallowing disorders and pregnant women were excluded from the study. In all enrolled cases, CE was performed first (because artifacts induced by DBE may have been difficult to interpret by CE). The endoscopists knew the results of CE at the time of DBE.

***Capsule endoscopy procedure: ***All patients underwent Pill Cam SB capsule (Given Imaging, Yoqneam, Israel) examination. The patients underwent bowel preparation with 2 L to 4 L of polyethylene glycol solution and fasted overnight, at least eight hours before the procedure. The capsule passed naturally through the gastrointestinal tract and took images of the intestine at a speed of two frames per second. The images were transmitted to the sensor array and saved to the data recorder. All equipment was disconnected after eight hours. Images were downloaded and reviewed by two independent experienced reviewers. The location of any lesion in the small bowel was determined by the transit time ratio. The patients were instructed to carefully check their stools in order to make sure that the capsules were eliminated from their bodies.

***Double-balloon enteroscopy procedure: ***The Fujinon DBE system (Fuji Photo Optical Incorporated Company, Fujinon Inc, Japan) was used. There are two types of endoscopes, the EN-450P5 type for general use and the EN-450T5 type for treatment. The general-use type is thinner with an external diameter of 8.5 mm and a forceps channel diameter of 2.2 mm. The therapeutic endoscope has an external diameter of 9.4 mm and a forceps channel of 2.8 mm. The patients were anaesthetized with 10 mL of oral 2% lidocaine hydrochloride before DBE through mouth, No specific preparation was required for the antegrade DBE besides fasting for 6-8 hours before the procedure. The retrograde route required bowel cleansing as in a colonoscopy. The techniques of insertion of DBE have been described elsewhere.^2^^,^^12^ Antegrade, retrograde or combined antegrade and retrograde DBEs were performed with or without intervention under conscious sedation or general anesthesia. Antegrade DBE was selected when time ratio of the lesion was less than 1/2- 2/3 and retrograde DBE was selected when time ratio of the lesion exceeded 1/2- 2/3 from CE. In patients without a definite lesion detected by CE, the route of insertion was determined according to clinical presentation. The DBE procedures were performed by two endoscopists (who did not interpret the CE findings) and assistants. Patients were observed in the recovery room or stayed overnight. Oxygen was inhaled with electrocardiography monitored when necessary.

DBE was not performed when the cause of small bowel disorders could be explained, the operation time was too long to be tolerated, and more than 1/2-2/3 of the small intestine examined was negative.

***Statistical analysis: ***Results are presented as median (range) for continuous data, and frequency (percentage) for categorical data. Statistical analysis was performed using SPSS 11.5. Differences were evaluated by using the Chi-square test. P < 0.05 were considered statistically significant.

**Fig.1:**
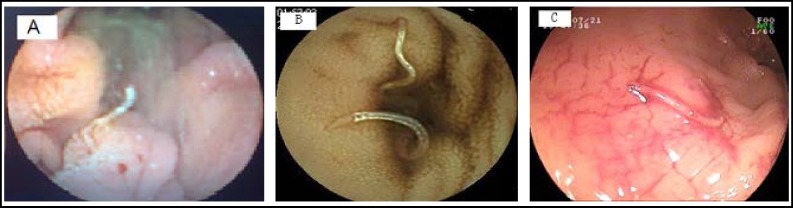
(A). Image of a suspected arteriovenous malformation as seen on capsule endoscopy in the proximal jejunum in a 36 years old male patient with obscure bleeding, which was revealed AVM by Double-balloon enteroscopy. (B). Image of two hookwarms as seen on capsule endoscopy in the distal ileum in a 55 years old female patient with unknown anemia, and (C). Double-balloon enteroscopy finding of many hookwarms in the ileum in the same patient

**Fig.2:**
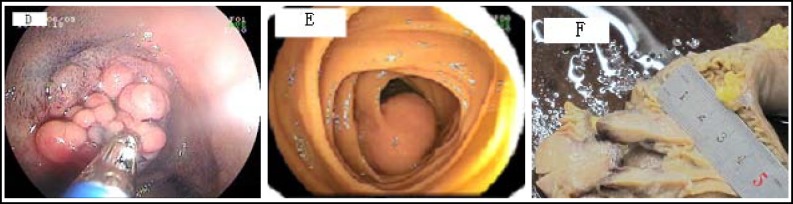
D). A multiple nodular polypoid lesion with a pink surrounding surface in the small bowel detected by double-balloon endoscopy, which revealed a Adenomatous polyp by pathology (The mild atypical hyperplasia). (E). Double-balloon enteroscopy, which revealed a great mass (3cm diameter) that was confirmed as a stromal tumor (malignant GIST) by surgery (F). Surgical gross specimen of malignant GIST (3cm diameter), the same patient

**Table-I:** Demographic data of 62 patients who underwent both capsule endoscopy and double-balloon enteroscopy

*Characteristic*	*Patients*
Age, years, mean (range)	55.4 (23–78)
Male:female	34:28
Type of small intestinal bleeding, n	33
Overt bleeding	10
Occult bleeding	23
Chronic abdominal pain,	11
Chronic diarrhea	9
Unexplained iron deficiency anemia	2
Other examinations showed abnormal small bowel imaging	2
A variety of inflammatory gastrointestinal disease	5

**Table-II:** Comparison of findings between CE and CE + DBE

*Finding*	*CE (n=62)*	*CE +DBE (n=62)*	*X* ^2^	*P*
Positive findings, n (%)	44 (70.9)	48(77.4)	0.6739	>0.05
Jejunum	25	28		
Ileum	19	20		
Bleeding, n (%)	26 (41.9)	30 (48.4)	0.5210	>0.05
Ulcer, n (%)	9 (14.5)	11 (17.7)	0.2384	>0.05
Angiodysplasia, n (%)	26 (41.9)	27 (43.5)	3.2952	>0.05
Angioma	1(1.61)	1(1.61)	0.5081	>0.05
Diverticulum	1(1.61)	1(1.61)	0.5081	>0.05
Abnormal mucosa	2(3.23)	3(4.84)	0	>0.05
Crohn’s disease	1(1.61)	1(1.61)	0.5081	>0.05
Mass, n (%)	4(6.45)	4(6.45)	0.1336	>0.05
Polyp, n (%)	2(3.23)	3(4.84)	0	>0.05
Malignancy, n (%)	1 ((1.61))	1 ((1.61))	0.5081	>0.05
Submucosal tumor, n (%)	1(1.61)	0		

**Table-III:** The small intestinal findings on double-balloon enteroscopy (DBE) in patients with negative evaluation or suspected findings on capsule endoscopy (CE

*Clinical diagnosis*	*CE: negative* *DBE: definite diagnosis*	*CE: suspected diagnosis* *DBE: definite diagnosis*	*CE: suspected diagnosis* *DBE: negative*
Bleeding lesions		5	1
Isolated ulcer	2^#^	1^#^	
Angioma			1
Angiodysplasia	1	2	
Polyp	1^#^	1^#^	
Malignancy		1^#*^	
Submucosal tumor			1
*#: The lesion was confirmed by biopsy. * *: The lesion was confirmed by surgery.			

## RESULTS

The demographic data of the 62 patients who underwent both CE and DBE are summarized in Table-I. Numerous diagnostic procedures were performed before both of the two procedures. These included gastroscopy (62/62, 100%), colonoscopy (62/62, 100%), small bowel study (25/62, 40.3%), angiography (5/62, 8.06%), CT of the abdomen (30/62, 48.4%), Meckel scan (3/62, 4.84%) and red blood cell scan (1/62, 1.61%).All these procedures could not diagnose the cause of obscure small bowel diseases.

***Findings on capsule endoscopy: ***The CE procedures were performed successfully in 62 patients. The small bowel lesions identified are shown in Table-II. The mean recording time was 465 minutes (360－680 minutes). The mean time to pass the small intestine was 255 minutes (210－520 minutes). No retention of the capsule occurred in any of the patients. The overall detection rate of small bowel disease by CE was 70.9% (44/62) (Fig.1). Which is lower than that (87%) as Lewis B et al reported.^10^^,^^11^

***Findings on double-balloon enteroscopy following capsule endoscopy: ***The DBE procedures were performed successfully in 62 patients (Table-II), antegrade DBE was performed in 36 patients, retrograde in 20, and combined antegrade and retrograde in 6 (total of 68 DBEs), all after CE (Fig.2). The mean duration of DBE was 80 minutes (range 50 to 180 minutes). The overall detection rate of small bowel disease with CE+DBE was (77.4%, 48/62), Fluoroscopy was used during the DBE procedures. Forty-two procedures were performed under general anesthesia and 26 procedures were performed under conscious sedation (fentanyl or meperidine with midazolam). The small bowel lesions identified by CE+DBE are shown in Table-II.

The mean interval time between CE and DBE was 15 days (4－60 days). A comparison of all diagnostic findings of CE and CE +DBE are shown in Table-II. Positive findings (77.4%) detected by CE +DBE is higher than that (70.9%) by CE, but the difference was not significant (χ2=0.6739, P>0.05). Angiodysplasia (CE 41.9% and CE +DBE 43.5%, P>0.5), ulcers (CE 14.5% and CE +DBE 17.7%, P>0.5), bleeding lesions (CE 41.9% and CE +DBE 48.4%, P>0.5) and mass (CE 6.45% and CE +DBE 6.45%, P>0.5). Furthermore, the detection rate of small bowel diseases in patients with obscure small intestinal bleeding using CE +DBE (90.9%, 30/33) was superior to that of CE (78.8%, 26/33); χ^2^=1.8857, P>0.05.

The suspected findings by CE were confirmed by DBE combined with biopsy in six patients and further confirmed by surgery in one patients. The small intestinal findings on double-balloon enteroscopy (DBE) in patients with negative evaluation or suspected findings on capsule endoscopy (CE) were shown in Table-III, DBE detected four cases of small bowel lesions, which were missed by CE. However, CE also detected suspect lesions that were not confirmed by DBE in three patients.

***Tolerance and adverse events: ***The 62 patients swallowed the capsules by themselves and did not describe CE as uncomfortable. All patients having had a successful CE were willing to accept a second procedure if needed. No adverse events occurred during the detention period of the capsules, including hemorrhage, perforation, acute pancreatitis or other serious complications occurred. Nausea, vomiting, abdominal distension, and abdominal pain occurred in 11 patients during the CE procedure. However, these symptoms were transient and tolerable. 

No procedure-related complications were observed in patients with a successful DBE. Twenty-eight patients (45.2%, 28/62) reported dizziness, light pharyngalgia, distention, light abdominal pain, nausea, or vomiting after DBE. The adverse reactions quickly resolved by themselves. In general, DBE through anus was more tolerable than through mouth.

## DISCUSSION

CE and DBE are both advanced inspection procedures of the small bowel for obscure small intestinal bleeding and obscure abdominal pain or diarrhea. They have common indications and quite different features. CE can cover the whole GI tract; the procedure requires no sedation^12^ and is better tolerated. Its major limitations are the inability to perform conventional endoscopic procedures such as air insufflation, rinsing, local re-examination, specimen biopsies and therapeutic interventions. Additionally, it may provide false-positive and false-negative findings due to its incontrollable movement and low-resolution pictures it takes. On the contrary, DBE was made possible to overcome these shortcomings.^8^^,^^9^^,^^13^^-^^15^ Moreover, endoscopic treatment procedures, including hemostasis, polypectomy, endoscopic mucosal resection, balloon dilation, and stent placement, can be performed.^16^ However, DBE is an invasive procedure that demands sedation or general anesthesia, fluoroscopic monitoring, the participation of two experienced endoscopists, and a prolonged examination time. a small number of adverse events have been reported (1%), such as pancreatitis and bowel perforation.^11^^,^^17^

Most study results showed that the diagnostic yields of both procedures were similar.^18^^-^^20^ Total small bowel examination by DBE usually requires a combination of the anterograde route and retrograde routes with a success rate of 42%~86%.^9^^,^^11^^,^^17^ The detection rate of small bowel abnormalities by DBE is limited by disturbances caused by air insufflations in the procedure or by failure to reach the lesion. However, CE may fail to identify lesions in the distal ileum due to dyskinesis of the gastrointestinal tract or disturbance by residual food or battery expiration.

The meta-analysis indicated that the yield of CE was higher compared to DBE with a single insertion approach, but might be lower than that of DBE with a combination of oral and anal approaches.^21^ However, no difference in the diagnostic yield with these two modalities was found in most studies.^18^^,^^19^ CE and DBE alone have their own advantages and limitations, It is therefore reasonable to use them in combination with each other to improve the detection rate and diagnostic accuracy of small bowel diseases. Accurate localization of the lesion in the small intestine by CE may facilitate the selection of the insertion route for DBE and decrease the number and time of examinations.

In the present study, we used the time ratio of the lesion detected by CE to estimate the site of the lesion in the small bowel under the assumption that CE travels at a constant velocity in the small bowel. Therefore, it is reasonable to select the antegrade route if the estimated site of the lesion is within the proximal one-half to two-thirds of the entire small bowel, i.e. time ratio of the lesion is less than 1/2-2/3; vice versa.

The CE procedures were performed successfully in 62 patients. The overall detection rate of small bowel disease by CE was 70.9% (44/62), which was slightly lower than the rate reported by LI Xiao-bo et al (72.0%).^22^ The DBE procedures were performed successfully in 62 patients, antegrade DBE was performed in 36 patients, retrograde in 20, and combined antegrade and retrograde in 6 (total of 68 DBEs), all after CE. The overall detection rate of small bowel disease with CE+DBE was (77.4%, 48/62), which was higher than the rate reported with DBE by LI Xiao-bo et al (41.2%).^22^ The suspected findings by CE were confirmed by DBE combined with biopsy in 6 patients and further confirmed by surgery in one patients. A comparison of all detection findings of CE and CE +DBE are nearly the same proportion of patients (70.9% versus 77.4%, respectively; P>0.05). This difference was not significant in the study. Unfortunately, the sample size in this study was not large enough to lead to an absolute conclusion; we expect that this difference will tell us more if we enlarge the sample size.

DBE detected four cases of small bowel lesions, which were missed by CE. Also, CE detected suspect lesions that were not confirmed by DBE in three patients.

## CONCLUSION

In conclusion, our study showed that CE and DBE are both effective modalities for diagnosis of small bowel diseases. CE can cover the whole GI tract and provide the selection of the route of DBE. DBE can also serve as a good complementary approach after an initial diagnostic imaging using CE. A capsule-directed DBE procedure might be better tolerated. DBE can verify the findings of CE and provide therapeutic intervention. Combined use of CE and DBE is effective in the diagnosis and management of patients with obscure small bowel diseases.

## Author’s contribution:

***Chen Tian Min: ***Endoscopy procedure, designing of the study. ***Xu Li Hua:*** Conceiving the idea, designing of the study. ***Ji Ying Lin:*** Collection of data, Identification picture of capsule endoscopy, coordination with hospital administration and getting consent from the patients. ***Yang Yan Mei:*** Statistical analysis and interpretation of the data. ***Lu Fei:*** Follow up of the patients. ***Qian Jun Bo: ***Endoscopy procedures, (double-balloon enteroscopy and capsule endoscopy).
